# COVID-19 cluster surveillance using exposure data collected from routine contact tracing: The genomic validation of a novel informatics-based approach to outbreak detection in England

**DOI:** 10.1371/journal.pdig.0000485

**Published:** 2024-04-25

**Authors:** Simon Packer, Piotr Patrzylas, Iona Smith, Cong Chen, Adrian Wensley, Olisaeloka Nsonwu, Kyle Dack, Charlie Turner, Charlotte Anderson, Rachel Kwiatkowska, Isabel Oliver, Obaghe Edeghere, Graham Fraser, Gareth Hughes

**Affiliations:** United Kingdom Health Security Agency, London, United Kingdom; University of Liverpool, UNITED KINGDOM

## Abstract

**Contact tracing was used globally to prevent onwards transmission of COVID-19. Tracing contacts alone is unlikely to be sufficient in controlling community transmission, due to the pre-symptomatic, overdispersed and airborne nature of COVID-19 transmission. We describe and demonstrate the validity of a national enhanced contact tracing programme for COVID-19 cluster surveillance in England.** Data on cases occurring between October 2020 and September 2021 were extracted from the national contact tracing system. Exposure clusters were identified algorithmically by matching ≥2 cases attending the same event, identified by matching postcode and event category within a 7-day rolling window. Genetic validity was defined as exposure clusters with ≥2 cases from different households with identical viral sequences. Exposure clusters were fuzzy matched to the national incident management system (HPZone) by postcode and setting description. Multivariable logistic regression modelling was used to determine cluster characteristics associated with genetic validity. **Over a quarter of a million (269,470) exposure clusters were identified. Of the eligible clusters, 25% (3,306/13,008) were genetically valid. 81% (2684/3306) of these were not recorded on HPZone and were identified on average of one day earlier than incidents recorded on HPZone. Multivariable analysis demonstrated that exposure clusters occurring in workplaces (aOR = 5·10, 95% CI 4·23–6·17) and education (aOR = 3·72, 95% CI 3·08–4·49) settings were those most strongly associated with genetic validity.** Cluster surveillance using enhanced contact tracing in England was a timely, comprehensive and systematic approach to the detection of transmission events occurring in community settings. Cluster surveillance can provide intelligence to stakeholders to support the assessment and management of clusters of COVID-19 at a local, regional, and national level. Future systems should include predictive modelling and network analysis to support risk assessment of exposure clusters to improve the effectiveness of enhanced contract tracing for outbreak detection.

## Author summary

We studied how to use routine contact tracing data to detect outbreaks of COVID-19. We identified clusters of people who had potentially been exposed to the virus at the same location (an exposure cluster). We then linked these to genomic data to identify whether clusters included persons who were infected with the same strain of the virus and therefore represent a shared transmission event. Our study is the first to show that routine contact tracing data can be used to systematically detect outbreaks at a population level. Using data from over 4 million cases of COVID-19 in England, we identified more than a quarter of a million exposure clusters. When linked to genomic data, we found that 25% of these were likely to be part of a real outbreak. The size and setting of these exposure clusters determined the likelihood that they were real outbreaks. We recommend that future responses take a machine learning approach to help them to prioritize resources and target their responses to signals that are more likely to represent real outbreaks. Overall, our study suggests that exposure cluster surveillance is a valuable tool for finding and controlling outbreaks of COVID-19 during a pandemic.

## Introduction

Globally, contact tracing was deployed during the COVID-19 pandemic to limit and prevent viral transmission through the identification and isolation of persons at greatest risk of developing disease [1–4]. It was recognised early in the pandemic that SARS-CoV-2 could be transmitted prior to symptom onset, and that transmission was overdispersed, with a minority of cases contributing to the majority of onward transmission events [5,6]. These observations suggested that conventional contact tracing alone, primarily focusing on the identification and isolation of named contacts, would have limited impact on the control of community transmission [2,7–13]. These observations were supported by several investigations of COVID-19 clusters where the primary cases and subsequent chains of transmission would not have been identified using traditional contact tracing methods alone [14–21]. While cluster surveillance based on confirmed cases can have significant utility (e.g. for the monitoring of continuing outbreaks in institutional settings), it provides at best indirect evidence for primary events responsible for transmission.

Backwards contact tracing (BCT) aims to identify the index case and other cases linked to the common source/setting of infection [22]. Modelling studies suggested that capturing cases’ exposure data during BCT could substantially increase contact tracing effectiveness [6,23]. This was supported by a prospective epidemiological study demonstrating the use and benefit of BCT among students in the Belgium [24]. A small number of countries, including Japan, adopted this approach early in the pandemic, leading to more timely recognition and termination of transmission chains [25,26].

In June 2020 the United Kingdom Scientific Advisory Group for Emergencies (SAGE) recommended that a bidirectional approach to contact tracing be developed and implemented [27]. The Public Health England (now United Kingdom Health Security Agency; UKHSA) Enhanced Contact Tracing Programme (ECT) was integrated with the conventional forwards national tracing programme of NHS Test and Trace (NHS T&T) in October 2020 and continued until the cessation of contact tracing of all cases in February 2022. The ECT programme deployed a cluster surveillance system based on case exposures during the pre-symptomatic period (3 to 7 days prior to symptom onset). Exposure data were collected on all cases during routine contact tracing and on a daily basis were algorithmically matched to data from other cases to define “exposure clusters”. These clusters were risk assessed by local public health teams to identify events and/or locations potentially associated with transmission [28]. We describe here the ECT programme in England, the epidemiology of clusters of case exposures of COVID-19 and provide evidence for the validity and operational utility of this approach for disease control through the early identification of transmission events and outbreaks.

## Methods

### COVID-19 contact tracing in England

The NHS T&T national contact tracing programme was launched in England in May 2020. The system received reports on all confirmed cases of SARS-CoV-2 identified through laboratory testing in England. Cases were initially invited by text message or email to self-complete a contact tracing questionnaire; those that couldn’t be contacted or who did not respond within a defined period were contacted by telephone [28,29]. Case information collected included demographic and clinical data, locations visited outside the home, and close contacts during the infectious period (defined as two days before symptoms or confirmatory laboratory test, to date of self-report of contact).

### Enhanced contact tracing in england

**Fig 1:**
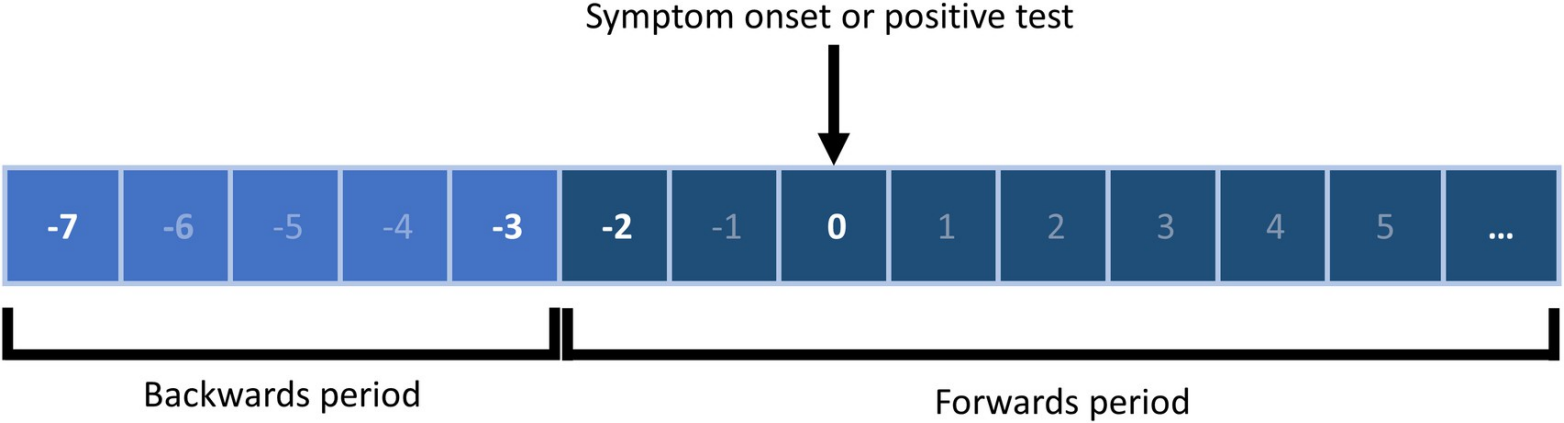
Periods of data collection for enhanced contact tracing. The backwards period of contact tracing was that likely to reflect probable exposure for the case (3–7 day period before symptom onset or date of the positive test). Data was collected on events and activities at workplace, education, household and other settings (such as hospitality and leisure).

Additional questions were added to the case questionnaire in October 2020 to collect information on events and activities outside the home during the period where infection was most likely to have occurred. This was defined as 3–7 days before symptom onset or date of positive test (Fig 1) [30]. Data collected included: event description, event category, attendance date, postcode, and proximity risk indicators (crowded, close contact, closed space). Event categories were defined at three levels: the first indicated the type of event (workplace/education, household or accommodation, or events/activities) while the second and third level categories provided an increasing level of detail regarding the type of activity and its location (S1 Table).

### Exposure clusters, 2-days window and same day event groupings

Exposure clusters were defined as instances where ≥2 cases reported attending an event with the same postcode and setting category (the location) and with attendance dates within a seven-day rolling period (for example, three exposure events would be linked together if cases attended the same location on the first, fourth and tenth day of the month). A 2-day event window was defined where matching events occurred within a two-day rolling window. Same day events were defined as where the matching events occurred on the same day.

### Ethical statement

Ethical approval was not required as the work was part of the public health response to COVID-19. Consent was not required as all data were originally collected for contact tracing and health protection purposes and fall under Regulation 3 of the UK Health Service (Control of Patient Information) Regulations 2002.

### Operational use of exposure cluster reports

Daily lists of exposure clusters were automatically processed into a PowerBI dashboard where they were accessed by local and regional public health teams and national incident managers, with regional-level access controlled using Microsoft security groups. Public health teams used exposure cluster information to identify, and risk assess clusters as possible outbreaks. Weekly surveillance reports on incident exposure clusters at local authority level were also made available to public health teams.

### Data analysis

#### Exposure clusters

Exposure clusters were identified from event data collected from confirmed cases of COVID-19 referred to NHS T&T for contact tracing between 23 October 2020 and 1 August 2021. Cases were included if they had completed a case questionnaire (via digital self-report or call handler), had a residential address in England, and reported at least one event outside their home. Events were linked to an exposure cluster if they matched deterministically on event postcode and setting category and had attendance dates within 7 days of another matched event. Matches were not permitted between events reported by the same case, e.g., if a case attended a workplace across multiple days. The notification date for exposure clusters was the date of entry of the second case into the contact tracing system. Exposure cluster reports were derived through daily linkage of all events reported by cases in the backwards period with geographical information and an attendance date within the past 30 days. Common exposures with a postcode outside England were removed.

#### Descriptive epidemiology

National case numbers by specimen date and vaccination data were obtained from the Public Health England (PHE) Coronavirus dashboard [31]. Descriptive analysis included trend analysis of events per case and frequency of exposure clusters by setting, number of cases, distribution of cases over time, background incidence, median age, duration, cumulative 2^nd^ dose vaccination coverage and sex ratio of the exposure cluster. Background incidence (cases per 100,000 population) and 2^nd^ dose vaccine coverage were assigned to exposure clusters based on the earliest attendance date and upper tier local authority (a local government structure responsible for a range of services to the population of a defined area) of the setting. Descriptive statistics (mean, median, interquartile range) were calculated according to the type of data. Events and exposure clusters were grouped into time periods based on the national restrictions in place in England [32,33].

#### Validation of exposure clusters using genomics data

Contact tracing records were linked to their corresponding laboratory records and whole genome sequencing data as previously described [34]. Exposure clusters were included in validation analysis if ≥2 cases were successfully linked to genomics data. An exposure cluster was considered genetically valid if it included ≥2 cases from different households where sequences were zero single-nucleotide polymorphisms apart. Household sharing was determined using unique property reference number (UPRN) obtained from address matching using the Ordnance Survey Address Base [35].

#### Exposure clusters and reported outbreaks and incidents

Data on COVID-19 incidents and outbreaks notified to and/or managed by regional health protection teams (HPT) were obtained from HPZone, the national health protection case and incident management system. Exposure clusters were linked to reported incidents/outbreaks by postcode and a further fuzzy match on the free text description of the exposure cluster setting provided by cases during contact tracing and included on HPZone. A successful match was made where ≥70% of words (a pragmatic cut-off, irrespective of length) matched between the exposure cluster and HPZone free text description (i.e., 70% of the words within each description were also found in the other description). Valid links were defined as those where the exposure cluster report date was up to seven days before or after the date the situation was entered onto HPZone.

#### Multivariable analysis

Single variable and multivariable analyses were used to identify factors associated with genetically valid exposure clusters. Odds ratios (OR) and corresponding 95% confidence intervals (CI) were calculated. A forward approach was used to build a model with the contribution of variables assessed through reduction of Akaike information criterion (AIC) and significance of likelihood ratio test (p<0.05). Variable coefficients and p-values were assessed in the single variable analysis and sequentially added to the multivariable model in order of decreasing significance.

Characteristics of exposure clusters considered for inclusion were the total number of cases, setting, median age of cases, number of same day events, duration, and standard deviation of the sex ratio. *A priori* confounders (background COVID-19 incidence, cumulative 2^nd^ dose vaccination coverage, urban-rural classification, [36] Index of Multiple Deprivation [37] of exposure postcode) significant through single variable analysis (p<0.05) were considered for inclusion in the model containing exposure cluster characteristics and effects assessed for inclusion as above.

The model was assessed for influential variables (Cook’s distance via residuals versus leverage plot using a cut off 0.5), multi-collinearity (variance inflation factor >5), and the assumption of linearity for continuous variables (Local Polynomial Regression Fitting via graphical assessment of “Loess” line monotonicity). Model prediction of genetic validity was assessed by calculating predicted probabilities and using a receiver operator curve (ROC) statistic. All analysis was undertaken using R version 4.2.1 [38].

### Role of the funding source

This work was conducted as part of the public health response to the COVID-19 pandemic in England.

## Results

### Contact tracing data and reported events

There were 4,628,798 confirmed cases referred for contact tracing during the study period, of which 89% (4,119,630) completed the contact tracing questionnaire. Of those, 57% (2,318,450) declared at least one event outside the home during the backwards period; these cases reported a total of 7,368,666 events (mean 1·6 events per case). Work or education events were most frequently reported with 4,474,540 events declared by 1,494,773 cases (average of 1·7 events per case; Table 1). The median interval between the earliest backwards event and symptom onset was 5 days, with a duration between onset and testing of 6 days and time to referral for contact tracing of 8 days (Table 1).

**Table 1:** Events declared by cases during contact tracing by direction relative to symptom onset and by event type.

Direction	Event Type	Cases	Total events	Mean events per case	Median events per case	Max events attended by a case	Median days between onset and exposure
Backward	Activity	1,050,092	2,140,285	0.8	1	60	5
	Household	247,827	753,841	0.3	3	19	5
	Work/education	1,494,773	4,474,540	1.7	3	43	5
Forwards	Activity	1,038,381	1,547,647	0.6	1	38	1
	Work/education	1,199,758	1,247,131	0.5	1	30	0
All events	..	2,679,077	10,163,444	3.8	3	63	4

### Epidemiology of exposure clusters in England

**Fig 2:**
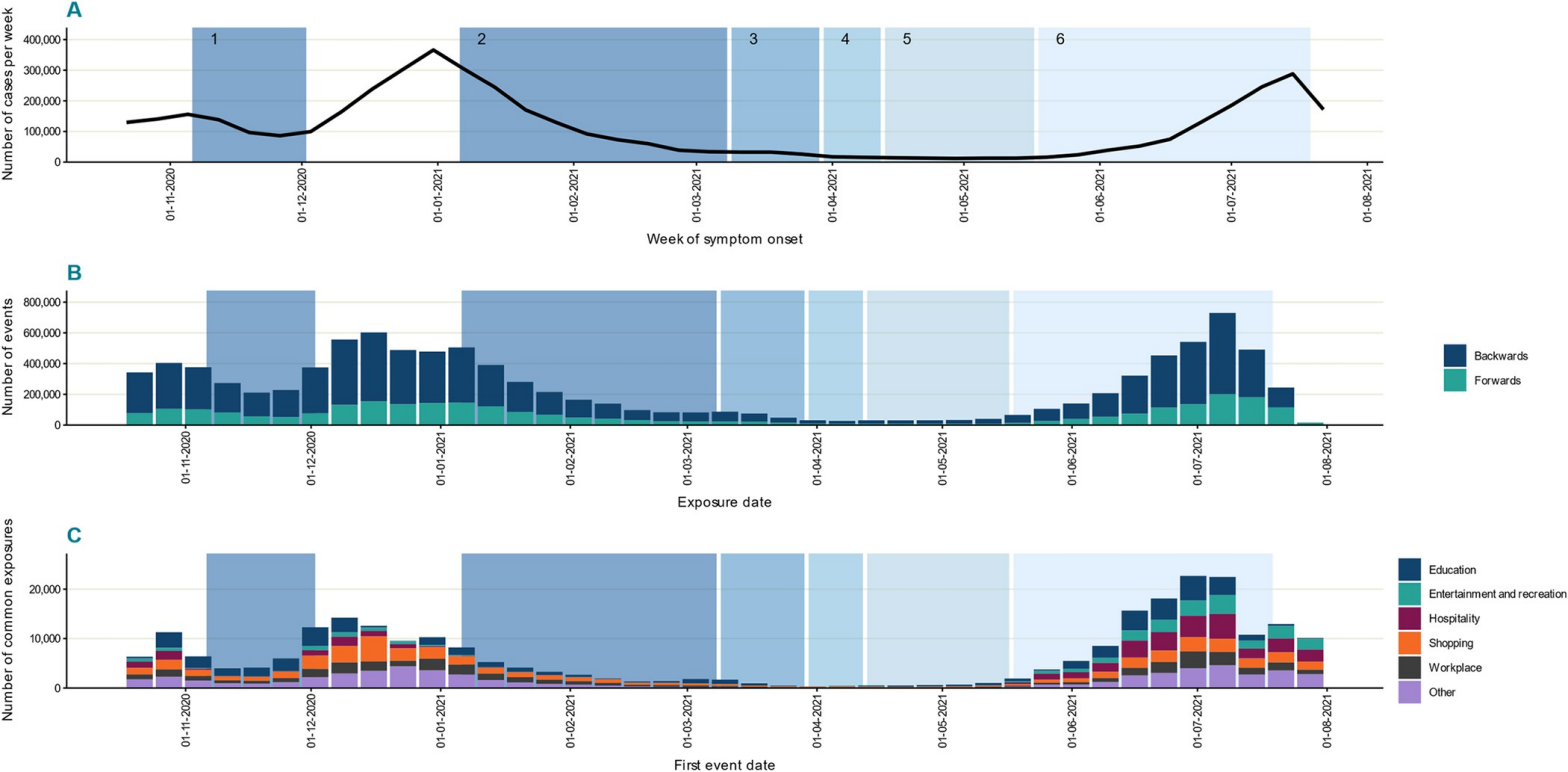
COVID-19 incidence and events and exposure clusters reported to contact tracing. (A) incidence of new confirmed cases; (B) number of events by period of attendance; (C) exposure clusters by event type. Backwards events reflect those reported by cases during the likely exposure period (3–7 days before symptom onset or date of positive test), forwards events those reported after the case was likely infectious (from 2 days before symptom onset or date of positive test to the time of contact tracing). Data is shown relative to national restrictions in England from 23 October 2020 to 1 August 2021. National non pharmaceutical interventions: 1: second national lockdown; 2: third national lockdown; 3–6: roadmap out of restrictions.

The distribution and magnitude of exposure clusters varied in relation to changes in case incidence and the implementation of national non-pharmaceutical interventions (Fig 2). Overall, more than a quarter of a million exposure clusters (269,470) were identified during the study period; a median of 4,142 (IQR: 1,402–10,598) clusters each week in England. At the peak, 22,879 exposure clusters were identified in a single week (July 12–18 2021). Clusters were most frequently identified in education (19·8%), shopping (19·4%) and workplace (14·3%) settings (Fig 2).

At the start of the study period there was a rapid increase in exposure clusters with a peak at the beginning of November 2020, closely following the peak of the concurrent wave of COVID-19 in England. Most of these exposure clusters were in education, hospitality, and entertainment settings. The lockdown period that followed in November 2020 was associated with a sharp but slightly delayed decrease in exposure clusters, with clusters in education settings retaining a high frequency. Lifting of the lockdown in December 2020 led to a substantial increase in exposure clusters: exposure cluster incidence was high in education settings, but also increased substantially in shopping, workplace and hospitality and entertainment settings. The increase was sustained throughout December 2020, with the exception of educational settings where exposure cluster numbers decreased following school and university closures.

The start of the next national lockdown in January 2021 was associated with a decrease in exposure cluster incidence, with declines in hospitality and entertainment settings, but not in workplace and other settings. From February 2021 to the end of May 2021, case numbers fell markedly, and exposure clusters remained infrequent. In June 2021 during the final lifting of national restrictions, case numbers rose sharply and a concomitant increase in exposure clusters was observed in all settings, with high numbers identified in hospitality, entertainment, education, and workplace settings (Fig 2).

### Factors associated with genetically valid exposure clusters

There were 13,058 (5·2%) exposure clusters eligible for inclusion in the analysis of genetic validity. Of these, 25% (3,306) were defined as genetically valid (Table 2). The proportion of genetically valid clusters varied over the study period: from 14% in November 2020 and July 2021 to 36% in April 2021. The proportion of genetically valid exposure clusters was highest in clusters of ≥10 cases (37%, 260/712), in education (35%, 1246/3528) or workplace (42%, 577/1371) settings and those containing greater than five instances where ≥2 cases reported attending on the same day (same day attendance) (43%, 470/1088) (Table 3). IMD and rural/urban classification were both not significantly associated with genetic validity in single variables analysis and were not included in multivariable modelling.

**Table 2:** Exposure cluster genetic validity and matching to managed incidents.

Genetic validity*	Number of cases (row %) (column %)		
	Matched†	Not matched	Total
Valid	622 (18.8%)(47.2%)	2684 (81.2%)(23.0%)	3306 (100.0%)(25.4%)
Invalid	696 (7.2%)(52.8%)	9006 (92.8%)(77.0%)	9702 (100.0%)(74.6%)
Total	1318 (10.1%)(100.0%)	11690 (89.9%)(100.0%)	13008 (100.0%)(100.0%)

*An exposure cluster was considered genetically valid if it included ≥2 cases from different households where sequences were zero single-nucleotide polymorphisms apart.

†Exposure clusters were matched to incidents reported on the national incident management by postcode and fuzzy matching with text descriptions of the setting.

**Table 3:** Crude and adjusted associations between characteristics of exposure clusters and genetic validity.

	Genetic validity*		Single variable analysis	Multivariable analysis	
Characteristic	Invalid (n = 9,745)	Valid (n = 3,313)	cOR (95% CI)	aOR (95% CI)	p-value
**Background COVID-19 incidence** †					
0–100	4,395 (70)	1,892 (30)	1.80 (1.55 to 2.09)	1.82 (1.55 to 2.14)	<0.001
101–200	2,088 (74)	716 (26)	1.43 (1.22 to 1.69)	1.35 (1.14 to 1.61)	<0.001
201–300	1,040 (81)	249 (19)	Ref.	Ref.	..
301–400	810 (82)	180 (18)	0.93 (0.75 to 1.15)	1.06 (0.85 to 1.32)	0.624
>401	1,191 (82)	255 (18)	0.89 (0.74 to 1.09)	0.93 (0.76 to 1.14)	0.492
Unknown	178	14			
**Total cases in exposure cluster**					
2	3,749 (76)	1,162 (24)	Ref.	Ref.	..
3–4	3,291 (75)	1,092 (25)	1.07 (0.97 to 1.18)	1.13 (1.00 to 1.27)	0.048
5–6	1,344 (75)	460 (25)	1.10 (0.97 to 1.25)	1.13 (0.95 to 1.34)	0.182
7–8	585 (73)	211 (27)	1.16 (0.98 to 1.38)	1.11 (0.88 to 1.39)	0.392
9–10	281 (70)	121 (30)	1.39 (1.11 to 1.73)	1.31 (0.99 to 1.74)	0.06
>10	452 (63)	260 (37)	1.86 (1.57 to 2.19)	1.63 (1.27 to 2.09)	<0.001
**Exposure cluster setting**					
Shopping	2,100 (90)	228 (9.8)	Ref.	Ref.	..
Custodial institutions	5 (83)	1 (17)	1.84 (0.10 to 11.5)	-	0.945
Education	2,282 (65)	1,246 (35)	5.03 (4.32 to 5.87)	3.72 (3.09 to 4.50)	<0.001
Entertainment and recreation	1,224 (82)	270 (18)	2.03 (1.68 to 2.46)	2.33 (1.91 to 2.86)	<0.001
Healthcare	190 (70)	82 (30)	3.98 (2.96 to 5.31)	3.09 (2.27 to 4.19)	<0.001
Hospitality	1,556 (76)	480 (24)	2.84 (2.40 to 3.37)	2.89 (2.41 to 3.47)	<0.001
Other	287 (77)	87 (23)	2.79 (2.11 to 3.67)	2.7 (2.02 to 3.58)	<0.001
Personal care	19 (73)	7 (27)	3.39 (1.31 to 7.81)	1.74 (0.48 to 4.92)	0.338
Public and large events	255 (84)	50 (16)	1.81 (1.28 to 2.50)	2.01 (1.40 to 2.85)	<0.001
Residence (own or other)	318 (75)	107 (25)	3.10 (2.39 to 4.00)	2.98 (2.25 to 3.93)	<0.001
Social care	151 (77)	46 (23)	2.81 (1.95 to 3.98)	2.4 (1.64 to 3.46)	<0.001
Travel	485 (81)	114 (19)	2.16 (1.69 to 2.76)	2.02 (1.55 to 2.63)	<0.001
Workplace	794 (58)	577 (42)	6.69 (5.63 to 7.97)	5.11 (4.24 to 6.18)	<0.001
Worship	36 (77)	11 (23)	2.81 (1.35 to 5.43)	2.59 (1.22 to 5.12)	0.008
**Median age of cases (years)**					
0–18	2,273 (70)	969 (30)	1.31 (1.14 to 1.51)	0.73 (0.62 to 0.87)	<0.001
19–25	2,120 (78)	596 (22)	0.87 (0.75 to 1.01)	0.91 (0.77 to 1.08)	0.271
31–35	1,596 (77)	486 (23)	0.94 (0.80 to 1.10)	0.92 (0.78 to 1.09)	0.323
26–30	1,112 (75)	361 (25)	Ref.	Ref.	..
36–40	967 (76)	301 (24)	0.96 (0.80 to 1.14)	0.96 (0.79 to 1.16)	0.675
41–50	1,130 (73)	421 (27)	1.15 (0.98 to 1.35)	1.13 (0.94 to 1.35)	0.183
>51	504 (75)	172 (25)	1.05 (0.85 to 1.30)	1.13 (0.90 to 1.41)	0.305
**Number of same day events**					
0–1 clusters	5,894 (82)	1,286 (18)	Ref.	Ref.	..
2 clusters	1,192 (73)	433 (27)	1.66 (1.47 to 1.89)	1.58 (1.37 to 1.81)	<0.001
3 clusters	904 (67)	440 (33)	2.23 (1.96 to 2.54)	1.88 (1.61 to 2.19)	<0.001
4–5 clusters	1,094 (62)	677 (38)	2.84 (2.53 to 3.18)	2.36 (2.03 to 2.75)	<0.001
>5 clusters	618 (57)	470 (43)	3.49 (3.05 to 3.99)	3.55 (2.87 to 4.39)	<0.001
**Exposure cluster duration (days)**					
0–1	1,383 (77)	420 (23)	Ref.	Ref.	..
2–4	2,221 (74)	762 (26)	1.13 (0.99 to 1.30)	0.62 (0.53 to 0.73)	<0.001
5–7	2,493 (76)	797 (24)	1.05 (0.92 to 1.21)	0.61 (0.52 to 0.71)	<0.001
8–10	1,821 (71)	733 (29)	1.33 (1.15 to 1.52)	0.55 (0.46 to 0.66)	<0.001
>10	1,784 (75)	594 (25)	1.10 (0.95 to 1.27)	0.39 (0.32 to 0.48)	<0.001
**Vaccination coverage (%)** ‡					
<25	3,963 (69)	1,798 (31)	Ref.	Ref.	..
25–50	3,527 (77)	1,041 (23)	0.65 (0.60 to 0.71)	0.69 (0.62 to 0.76)	<0.001
51–75	2,037 (82)	453 (18)	0.49 (0.44 to 0.55)	0.61 (0.53 to 0.69)	<0.001
Unknown	175	14			
**Standard deviation of sex ratio**	0.62 (0.10–1.45)	0.72 (0.21–1.45)	1.22 (1.14 to 1.30)	1.2 (1.12 to 1.29)	<0.001
Unknown	24	4			
1OR = Odds Ratio, CI = Confidence Interval					

^1^n (%); Median (IQR); cOR = Crude Odds Ratio; CI = Confidence Interval; aOR = Adjusted Odds Ratio; NA = not applicable.

*Genetic validity was defined as an exposure cluster including two or more cases from different households where sequences were zero SNPs apart.

†Cases per 100,000 population on the start date of the exposure cluster in the upper tier local authority of the setting.

‡ Cumulative 2^nd^ dose vaccination coverage on the start date of the exposure cluster in the upper tier local authority of the common exposure setting.

The final model included 12,786 observations (267 exposure clusters excluded due to: postcodes outside of England, missing values for ≥1 variable or found to be highly influential on model fit) and had an area ROC of 0·71 (95% CI 0·70–0·72). Five influential observations were removed from the model which resulted in large percentage change in the association between two settings (personal care and custodial institutions) and genetic validity. No collinearity was observed between variables included in the final model. All continuous variables showed no substantial evidence of non-linearity through visual assessment.

Exposure clusters that included more cases, were shorter in duration, and contained a greater number of same day events, were more likely to indicate genetically valid transmission events (Table 3). There was a dose-response relationship between the number of events in an exposure cluster and likelihood of genetically linked cases. Clusters of longer duration were significantly less likely to represent genetically valid signals for outbreaks. An increased number of same day events within the cluster was associated with genetically linked cases, with odds increasing significantly (using the absence of same day events as the reference group) with the number of same day events included: two same day events (aOR 1·58 [95% CI 1·37–1·82]) and >5 same day events (aOR 3·57 [95% CI 2·89–4·41]) (Table 3).

Clusters in all settings other than those in custodial institutions were found to be independently associated with increased odds of genetic validity (using shopping as the reference group). Strong associations were observed for workplace settings (aOR 5·10 [95% CI 4·23–6·17]), education settings (aOR 3·72 [95% CI 3·08–4·49]), healthcare settings (aOR 3·09 [95% CI 2·27–4·19]), and hospitality settings (aOR 2·89 [95% CI 2·41–3·47]).

### Genetically valid exposure clusters and reported incidents/outbreaks

Over 5% (13,494/248,864) of all exposure clusters identified during the study period were linked to incidents recorded on the national incident management system (HPZone). Of the exposure clusters eligible for inclusion in the genetic validity analysis (n = 13,008), 47% (622/1318) of HPZone matched exposure clusters were genetically valid compared to 23% (2684/11690) of those that were not matched. Genetically valid exposure clusters linked to situations on HPZone were identified a median of one day (IQR 0–4, range -7 to 7) earlier through ECT than the corresponding entry on HPZone (Table 2).

## Discussion

In this study we have described the epidemiology of COVID-19 case exposure clusters identified by the ECT programme in England and provided evidence for their validity and utility for the rapid identification of outbreaks. Approximately 25% of exposure clusters detected through the ECT programme included ≥2 genetically indistinguishable SARS-CoV-2 infections. This proportion increased to >30% during low incidence periods, where the impact of early action by local public health teams to break transmission chains would be highest. We have also identified cluster characteristics independently associated with increased likelihood of genetic validity; these include clusters of larger size, including same day events, and those in particular settings (including healthcare and workplaces).

The ECT cluster surveillance system frequently detected outbreaks before they were recorded as managed incidents by local health protection services; approximately one half of exposure clusters linked to subsequently confirmed outbreaks were detected before registration on the national incident management system. These events frequently occurred outside of formal institutional settings and could represent important foci of community transmission. Exposure cluster settings included hospitality and mass gatherings, where contacts were likely to be unknown to each other and would not be rapidly identified, if at all, through conventional contact tracing. Community settings contribute significantly to onward spread of COVID-19 [14,17,18,39–42] and cluster identification provided corroborative and real-time information to support local risk assessment and management of outbreaks.

To our knowledge, the ECT programme in England was the only national programme using contact tracing information for systematic surveillance of COVID-19 clusters based on the exposures of cases during their pre-symptomatic period. A key consideration for any cluster surveillance system is achieving the optimal balance between sensitivity and specificity. The ECT exposure cluster algorithm was initially designed to prioritise sensitivity over specificity and used a broad time period and postcodes for linking case events. We have shown that clusters defined through shorter time period linkages (e.g., 2-day event window or same day events) are more likely to represent actual transmission events and can be used to improve specificity. Furthermore, the use of unique property reference numbers was introduced towards the end of the programme to increase the precision of geolocation. Improving specificity whilst maintaining sensitivity would be a key development for future cluster surveillance.

The strengths of this study stems from the secondary analysis of systematically collected national contact tracing data. Exposure data was collected from more than 85% of confirmed COVID-19 cases in England over the study period, providing comprehensive and representative coverage with considerable statistical power. Linkage to available genomics data provided a means to validate exposure clusters using a highly specific indicator of probable transmission. Although genomic sequencing coverage limited the proportion of cases which could be included in assessment of genomic validity, cases were largely selected randomly for sequencing (by geographically weighted sampling of community cases), with some oversampling of some high-risk groups (such as healthcare workers and international travellers). Strengthening the coverage and timeliness of genomic surveillance is critical for more effective cluster detection of this kind.

Limitations include the use of primary source data collected for operational purposes, and likely subject to a degree of heterogeneity and incompleteness in data collection. A significant proportion of exposure events may not have been recorded because cases were either unaware or deliberately chose not to report them, although the direction and potential size of any resulting bias is unclear. Additionally, the genetic validity investigations were based on a small proportion of all exposure clusters, this may have introduced representativity bias, the nature and direction of which cannot be determined.

The use of a highly specific definition for genetic validity means we have likely underestimated the true number of valid clusters. Minor variant genomes can emerge to dominance within an individual [43] with the potential for genetic compartmentalisation between the respiratory tract and gastrointestinal tract [44]. In addition, treatments for COVID-19 that interfere with viral replication can induce mutational signatures associated with greater sequence divergence between transmission pairs [45]. Such signals may be greater in certain population groups (e.g., older adults more likely to receive treatment).

Given that transmission of a genetically identical sequence is more likely to occur earlier during an infection [43], settings more likely to be associated with close to continuous exposure (such as households) are more likely to have been detected using our conservative methodology. The observation that longer clusters were less likely to be genetically valid may also be in part due to the accumulation of substitutions during longer transmission chains. Future work needs to assess the impact of these elements and evaluate the use of more relaxed genetic matches on cluster assessment and outbreak detection.

The ECT programme identified and communicated exposure clusters to local public health teams daily. Based on expert opinion and guidance, exposure clusters were risk assessed for the need for public health action. Without the availability of genetic validation during the response, exposure clusters lacked specificity. In future we recommend that a predictive modelling approach, which uses genomic validation, is used to help triage and prioritise clusters for risk assessment. The use of predictive modelling and genomic validation could enable real-time model calibration based on changes in background epidemiology of the virus. However, such an approach may be limited by the turnaround time for sequencing of isolates. Further work could use network analysis methods to combine exposure cluster data with other available transmission indicators to build a transmission network of extant links. These networks could be used to infer the setting/source of infection for all cases as the pandemic progresses, providing vital information on which settings are associated with transmission and to target interventions.

For this study we employed simplistic text matching methods for detection of exposure clusters and linkage to situations under public health management. This has exposed requirements for machine learning methods to improve text matching and exposure cluster detection from contact tracing data. Additionally, these is a further need to develop unsupervised machine learning models to provide timely predictions that exposure clusters are outbreaks. These three health protection requirements for future work are detailed in Table 4.

**Table 4:** Technological development needs for future studies.

Development need	Potential methods
Accurate matching of events reported during contact tracing	Natural language processing (unsupervised machine learning) with clustering algorithms to identify patterns in events and outbreak data. Data parsed into manageable units and matched through application of a clustering algorithm (such as K-Means or topic modelling).
Accurate matching of exposure clusters to outbreaks under public health management	
Timely estimates for the probability that exposure clusters are real outbreaks	Unsupervised machine learning model using all available data (including public health management data and genomics data) to provide predictions that newly detected exposure clusters are outbreaks. Methodologies could include Bayesian networks and neural networks.

Through analysis of routine contact tracing data collected in England, we have shown that systematic case exposure cluster surveillance is a feasible and valid tool for outbreak detection and situational awareness that can complement traditional methods. Although an evaluation of the effectiveness of such programmes to reduce transmission are required, exposure cluster surveillance should be considered for pandemics or epidemics where contact tracing is integral to the response. The methodology may be applicable across a range of infectious diseases, particularly those characterised by overdispersion of transmission and where transmission occurs across a variety of different settings.

## Supporting information

S1 Table Event categories used to classify forward and backward events reported by confirmed COVID-19 cases in the national contact tracing system in England. (DOCX)
