# Supplementary material for: COVID-19 cluster surveillance using exposure data collected from routine contact tracing: The genomic validation of a novel informatics-based approach to outbreak detection in England
Source: PLOS Digit Health. 2024 Apr 25;3(4):e0000485. doi: 10.1371/journal.pdig.0000485 (PMC11045073; doi:10.1371/journal.pdig.0000485)
Supplement: S1 Table — (DOCX) [file pdig.0000485.s001.docx]

**S1 Table: Event categories used to classify forward and backward events reported by confirmed COVID-19 cases in the national contact tracing system in England.** Events data was collected in a structured manner with activities categorised by three nested levels providing increasing level of detail on activities. The highest level (Level 1) categorises the exposures to work and education activity, or leisure activities. Further information includes sectors, for example, educational, or hospitality setting. Level 3 is the most granular level.

| **Level 1** | **Level 2** | **Level 3** |
| --- | --- | --- |
| Events/ activities | Community and charity activities | Boot sale |
| Events/ activities | Community and charity activities | Charity event |
| Events/ activities | Community and charity activities | Communal hobbies |
| Events/ activities | Community and charity activities | Community event |
| Events/ activities | Community and charity activities | Corporate event |
| Events/ activities | Community and charity activities | Educational classes |
| Events/ activities | Community and charity activities | Fundraising |
| Events/ activities | Community and charity activities | Other |
| Events/ activities | Community and charity activities | Religious event |
| Events/ activities | Community and charity activities | Volunteering |
| Events/ activities | Eating out | Markets |
| Events/ activities | Eating out | Mobile Food Vendor |
| Events/ activities | Eating out | Other |
| Events/ activities | Eating out | Pub/Bar |
| Events/ activities | Eating out | Restaurant/Cafe - Café |
| Events/ activities | Eating out | Restaurant/Cafe - Chinese |
| Events/ activities | Eating out | Restaurant/Cafe - Chinese |
| Events/ activities | Eating out | Restaurant/Cafe - Coffee shop |
| Events/ activities | Eating out | Restaurant/Cafe - English |
| Events/ activities | Eating out | Restaurant/Cafe - French |
| Events/ activities | Eating out | Restaurant/Cafe - Fried chicken takeaway |
| Events/ activities | Eating out | Restaurant/Cafe - Indian |
| Events/ activities | Eating out | Restaurant/Cafe - Italian |
| Events/ activities | Eating out | Restaurant/Cafe - Kebab takeaway |
| Events/ activities | Eating out | Restaurant/Cafe - Other |
| Events/ activities | Eating out | Restaurant/Cafe - Sandwich shop |
| Events/ activities | Eating out | Restaurant/Cafe - Thai |
| Events/ activities | Entertainment and day trips | Amusement park |
| Events/ activities | Entertainment and day trips | Bingo |
| Events/ activities | Entertainment and day trips | Cinema |
| Events/ activities | Entertainment and day trips | Comedy club |
| Events/ activities | Entertainment and day trips | Festival |
| Events/ activities | Entertainment and day trips | Live Music |
| Events/ activities | Entertainment and day trips | Museum/ gallery |
| Events/ activities | Entertainment and day trips | Night Club |
| Events/ activities | Entertainment and day trips | Organised trips |
| Events/ activities | Entertainment and day trips | Other |
| Events/ activities | Entertainment and day trips | Park/playground |
| Events/ activities | Entertainment and day trips | Play groups/play date |
| Events/ activities | Entertainment and day trips | Pub/Bar |
| Events/ activities | Entertainment and day trips | Social club |
| Events/ activities | Entertainment and day trips | Soft play |
| Events/ activities | Entertainment and day trips | Theatre |
| Events/ activities | Entertainment and day trips | Zoo/aquarium/petting farm |
| Events/ activities | Exercising | Adventure sports |
| Events/ activities | Exercising | Casual exercise with friend(s) |
| Events/ activities | Exercising | Classes (e.g. Yoga, Pilates) |
| Events/ activities | Exercising | Clubs (e.g. running) |
| Events/ activities | Exercising | Gym |
| Events/ activities | Exercising | Indoor sports facility |
| Events/ activities | Exercising | Other |
| Events/ activities | Exercising | Outdoor sports facility |
| Events/ activities | Exercising | Swimming |
| Events/ activities | Personal care | Barbers and Hairdressers |
| Events/ activities | Personal care | Beauticians |
| Events/ activities | Personal care | Massage therapist |
| Events/ activities | Personal care | Nail bars |
| Events/ activities | Personal care | Other |
| Events/ activities | Personal care | Spa |
| Events/ activities | Personal care | Tattooists |
| Events/ activities | Private events and celebrations | Christening |
| Events/ activities | Private events and celebrations | Funeral |
| Events/ activities | Private events and celebrations | Gala/Reunions |
| Events/ activities | Private events and celebrations | Other |
| Events/ activities | Private events and celebrations | Party |
| Events/ activities | Private events and celebrations | Wedding |
| Events/ activities | Public events and mass gathering | Concerts |
| Events/ activities | Public events and mass gathering | Demonstration/Protest |
| Events/ activities | Public events and mass gathering | Festival |
| Events/ activities | Public events and mass gathering | Gig |
| Events/ activities | Public events and mass gathering | Other |
| Events/ activities | Public events and mass gathering | Rave |
| Events/ activities | Shopping | Bookshop |
| Events/ activities | Shopping | Clothes shopping |
| Events/ activities | Shopping | Department store |
| Events/ activities | Shopping | DIY Store |
| Events/ activities | Shopping | Garage |
| Events/ activities | Shopping | Garden Centre |
| Events/ activities | Shopping | Indoor/covered market |
| Events/ activities | Shopping | Local convenience store |
| Events/ activities | Shopping | Other |
| Events/ activities | Shopping | Outdoor market |
| Events/ activities | Shopping | Pharmacy |
| Events/ activities | Shopping | Supermarket – Named |
| Events/ activities | Shopping | Supermarket - Named |
| Events/ activities | Shopping | Supermarket - Named |
| Events/ activities | Shopping | Supermarket - Named |
| Events/ activities | Shopping | Supermarket - Named |
| Events/ activities | Shopping | Supermarket - Named |
| Events/ activities | Shopping | Supermarket - Named |
| Events/ activities | Shopping | Supermarket – Named |
| Events/ activities | Shopping | Supermarket – Named |
| Events/ activities | Shopping | Supermarket – Named |
| Events/ activities | Sport events | Boxing or martial arts |
| Events/ activities | Sport events | Cricket match |
| Events/ activities | Sport events | Cycling event |
| Events/ activities | Sport events | Football match |
| Events/ activities | Sport events | Hockey match |
| Events/ activities | Sport events | Horse races |
| Events/ activities | Sport events | Netball match |
| Events/ activities | Sport events | Other |
| Events/ activities | Sport events | Rugby match |
| Events/ activities | Sport events | Tennis match |
| Events/ activities | Travel and commuting | Car share |
| Events/ activities | Travel and commuting | Coach - domestic |
| Events/ activities | Travel and commuting | Coach - international |
| Events/ activities | Travel and commuting | Cruise/ship |
| Events/ activities | Travel and commuting | Eurostar/International train |
| Events/ activities | Travel and commuting | Flight - domestic |
| Events/ activities | Travel and commuting | Flight - international |
| Events/ activities | Travel and commuting | Other |
| Events/ activities | Travel and commuting | Other small vehicles |
| Events/ activities | Travel and commuting | Public transport - bus |
| Events/ activities | Travel and commuting | Public transport - other |
| Events/ activities | Travel and commuting | Public transport - underground/tram |
| Events/ activities | Travel and commuting | Taxi |
| Events/ activities | Travel and commuting | Train - domestic |
| Events/ activities | Visiting a health or social care setting for non-Covid 19 reasons | Care/Nursing home |
| Events/ activities | Visiting a health or social care setting for non-Covid 19 reasons | Community hospital |
| Events/ activities | Visiting a health or social care setting for non-Covid 19 reasons | Day centre |
| Events/ activities | Visiting a health or social care setting for non-Covid 19 reasons | Drop in clinic |
| Events/ activities | Visiting a health or social care setting for non-Covid 19 reasons | General Practice |
| Events/ activities | Visiting a health or social care setting for non-Covid 19 reasons | Hospital |
| Events/ activities | Visiting a health or social care setting for non-Covid 19 reasons | Other |
| Events/ activities | Visiting a health or social care setting for non-Covid 19 reasons | Pharmacy |
| Events/ activities | Visiting a health or social care setting for non-Covid 19 reasons | Residential home |
| Events/ activities | Visiting a military setting | - |
| Events/ activities | Visiting a prison / detention facility | - |
| Events/ activities | Visiting friends or relatives | Home |
| Events/ activities | Visiting friends or relatives | Other |
| Events/ activities | Visiting friends or relatives | Outdoor setting |
| Events/ activities | Worship and prayer | Church |
| Events/ activities | Worship and prayer | Dao guan |
| Events/ activities | Worship and prayer | Gurdwara |
| Events/ activities | Worship and prayer | Jinja |
| Events/ activities | Worship and prayer | Mosque |
| Events/ activities | Worship and prayer | Other |
| Events/ activities | Worship and prayer | Shrine |
| Events/ activities | Worship and prayer | Synagogue |
| Events/ activities | Worship and prayer | Temple |
| Household or accommodation | Holiday accommodation | Campsite |
| Household or accommodation | Holiday accommodation | Hotel/ B&B |
| Household or accommodation | Holiday accommodation | Other |
| Household or accommodation | Holiday accommodation | Private holiday house/flat |
| Household or accommodation | Holiday accommodation | Youth hostel |
| Household or accommodation | Other accommodation | Children's home |
| Household or accommodation | Other accommodation | Hostel |
| Household or accommodation | Other accommodation | Military block/ mess |
| Household or accommodation | Other accommodation | Other |
| Household or accommodation | Other accommodation | Prison |
| Household or accommodation | Other accommodation | Shelter |
| Household or accommodation | Shared accommodation | Other shared accommodation |
| Household or accommodation | Shared accommodation | Renting a place with people you don't know: 5 or more of you |
| Household or accommodation | Shared accommodation | Renting a place with people you don't know: up to 4 including you |
| Household or accommodation | Shared accommodation | Renting a place with people you know: 5 or more of you |
| Household or accommodation | Shared accommodation | Renting a place with people you know: up to 4 including you |
| Household or accommodation | Shared accommodation | Student halls of residence |
| Household or accommodation | Supported living | Self-contained flat |
| Household or accommodation | Supported living | Shared facilities (bathroom/ kitchen/ common room) |
| Household or accommodation | Your own home, or family home | Household of 5 or more people |
| Household or accommodation | Your own home, or family home | Household of fewer than 5 people |
| Household or accommodation | Your own home, or family home | Living alone |
| Work or education | Arts, entertainment or recreation | Cinema |
| Work or education | Arts, entertainment or recreation | Gym/ sports/ leisure facility |
| Work or education | Arts, entertainment or recreation | Museum/ gallery |
| Work or education | Arts, entertainment or recreation | Music |
| Work or education | Arts, entertainment or recreation | Other |
| Work or education | Arts, entertainment or recreation | Theatre/ Dance |
| Work or education | Attending childcare, school, educational setting | Childminder |
| Work or education | Attending childcare, school, educational setting | College |
| Work or education | Attending childcare, school, educational setting | Nursery/Preschool |
| Work or education | Attending childcare, school, educational setting | Other educational setting not listed |
| Work or education | Attending childcare, school, educational setting | Other Higher Education |
| Work or education | Attending childcare, school, educational setting | Primary School |
| Work or education | Attending childcare, school, educational setting | Secondary School |
| Work or education | Attending childcare, school, educational setting | Special needs educational setting |
| Work or education | Attending childcare, school, educational setting | University |
| Work or education | Civil service or Local Government | - |
| Work or education | Close contact services | Barbers and Hairdressers |
| Work or education | Close contact services | Beauty and nail bars |
| Work or education | Close contact services | Make-up studios |
| Work or education | Close contact services | Other close contact profession not listed |
| Work or education | Close contact services | Spas and wellness business |
| Work or education | Close contact services | Tanning salons or booths |
| Work or education | Close contact services | Tattooists |
| Work or education | Critical national infrastructure | - |
| Work or education | Emergency services | Ambulance service |
| Work or education | Emergency services | Fire service |
| Work or education | Emergency services | Other emergency service |
| Work or education | Emergency services | Police |
| Work or education | Financial services incl. insurance | - |
| Work or education | Food production and agriculture | Farming and agriculture |
| Work or education | Food production and agriculture | Food growing/ production (fruit/ vegetables/ animal products) |
| Work or education | Food production and agriculture | Food manufacture (abbatoir/ meat products) |
| Work or education | Food production and agriculture | Food manufacture (bakery/ confectionary) |
| Work or education | Food production and agriculture | Food manufacture (beverages) |
| Work or education | Food production and agriculture | Food manufacture (dairy products) |
| Work or education | Food production and agriculture | Food manufacture (fruit/ vegetables) |
| Work or education | Food production and agriculture | Food manufacture (ready meals) |
| Work or education | Food production and agriculture | Other food an agriculture profession not listed |
| Work or education | Health care | Ambulance service |
| Work or education | Health care | Community hospital |
| Work or education | Health care | Drop in clinic |
| Work or education | Health care | General Practice |
| Work or education | Health care | Hospital |
| Work or education | Health care | Other healthcare setting not listed |
| Work or education | Hospitality | Food and Drink (Restaurant, Bar, Pub, fast food outlet) |
| Work or education | Hospitality | Lodging (Hotel, B&B) |
| Work or education | Hospitality | Other hospitality profession not listed |
| Work or education | Hospitality | Tourism |
| Work or education | Immigration / border force services | Office based |
| Work or education | Immigration / border force services | People facing |
| Work or education | Information and communication | - |
| Work or education | Manufacturing or construction | Construction labour |
| Work or education | Manufacturing or construction | Manufacture (Cars) |
| Work or education | Manufacturing or construction | Manufacture (Chemical plant) |
| Work or education | Manufacturing or construction | Manufacture (Electronics) |
| Work or education | Manufacturing or construction | Manufacture (Engineering) |
| Work or education | Manufacturing or construction | Manufacture (Furniture) |
| Work or education | Manufacturing or construction | Manufacture (Pharmaceuticals) |
| Work or education | Manufacturing or construction | Manufacture (Printing) |
| Work or education | Manufacturing or construction | Manufacture (Textiles/ clothes) |
| Work or education | Manufacturing or construction | Office-based |
| Work or education | Manufacturing or construction | Other manufacturing/construction profession not listed |
| Work or education | Military (incl. civilian employees) | Air Force |
| Work or education | Military (incl. civilian employees) | Army |
| Work or education | Military (incl. civilian employees) | Navy |
| Work or education | Military (incl. civilian employees) | Other military profession not listed |
| Work or education | Other occupational sector | - |
| Work or education | Prison / detention facility | - |
| Work or education | Retail sector | Entertainment |
| Work or education | Retail sector | Fashion |
| Work or education | Retail sector | Food |
| Work or education | Retail sector | Health & beauty |
| Work or education | Retail sector | Home |
| Work or education | Retail sector | Newsagents |
| Work or education | Retail sector | Other retail sector profession not listed |
| Work or education | Retail sector | Sports & leisure |
| Work or education | Retail sector | Supermarket |
| Work or education | Retail sector | Technology |
| Work or education | Social care or home care | Care home |
| Work or education | Social care or home care | Domiciliary care |
| Work or education | Social care or home care | Health visitor |
| Work or education | Social care or home care | In home carer |
| Work or education | Social care or home care | Other social/home care setting not listed |
| Work or education | Teaching and education | Childminder |
| Work or education | Teaching and education | Nursery/Preschool |
| Work or education | Teaching and education | Other educational setting not listed |
| Work or education | Teaching and education | Other Higher Education |
| Work or education | Teaching and education | Primary School |
| Work or education | Teaching and education | Secondary School |
| Work or education | Teaching and education | Sixth Form Centre |
| Work or education | Teaching and education | Special needs educational setting |
| Work or education | Teaching and education | University |
| Work or education | Transport | Bus |
| Work or education | Transport | Logistics |
| Work or education | Transport | Other public transport not listed |
| Work or education | Transport | Storage |
| Work or education | Transport | Taxi |
| Work or education | Transport | Train |
| Work or education | Transport | Underground/Tram |
| Work or education | Warehouse or distribution | Food distribution |
| Work or education | Warehouse or distribution | Haulage |
| Work or education | Warehouse or distribution | Warehouse |
| Work or education | Warehouse or distribution | Wholesalers |
| Work or education | Work travel or activity outside workplace | Conference attendance |
| Work or education | Work travel or activity outside workplace | Door to door sales |
| Work or education | Work travel or activity outside workplace | Home care visits |
| Work or education | Work travel or activity outside workplace | Other work travel not listed |
| Work or education | Work travel or activity outside workplace | Site visits |
| Work or education | Work travel or activity outside workplace | Visiting a clients house |
